# Extraction of Antioxidant Components from *Bidens pilosa* Flowers and Their Uptake by Human Intestinal Caco-2 Cells

**DOI:** 10.3390/molecules18021582

**Published:** 2013-01-25

**Authors:** Lee Wen-Chin, Peng Chiung-Chi, Chang Chi-Huang, Huang Shiau-Huei, Chyau Charng-Cherng

**Affiliations:** 1 Division of Nephrology, Department of Internal Medicine, Show Chwan Memorial Hospital, Changhua 500, Taiwan; 2 Graduate Institute of Clinical Medicine, College of Medicine, Taipei Medical University, Xin-Yi District, Taipei 110, Taiwan; 3 Research Institute of Biotechnology, Hungkuang University, 34 Chung-Chie Road, Shalu District, Taichung 433, Taiwan

**Keywords:** *Bidens pilosa* L. var. *radiata*, chemical composition, antioxidant, human intestinal Caco-2 cell line, uptake, HPLC-MS

## Abstract

*Bidens pilosa* L. var. *radiata* (BPR, Asteraceae) is a commonly used folk medicine for treating various disorders such as diabetes, inflammation and hypertension. Recent studies to determine its chemical composition have revealed three di-*O*-caffeoylquinic acids (DiCQAs) and three polyacetylene glucosides (PGAs) to be among the major bioactive markers. To obtain the major compounds of these two chemical classes, the ethyl acetate fraction (EM) obtained using liquid-liquid partition from the methanol extract resulted in a fraction with the highest total phenolic and total flavonoid contents and antioxidant activities in radical scavenging and ferric reducing power assays. To assess the bioavailability of EM, we examined the *in vitro* uptake using the Caco-2 human colonic cell line. The apparent permeability coefficient (Papp) for each of the compounds within PGAs measured in both apical (AP) to basolateral (BL) and BL to AP was found to preferentially appear BL to AP direction, indicated that a basolateral to apical efflux system was detected in the study. DiCQAs had a lower efflux ratio than those from PGAs (2.32–3.67 *vs.* 6.03–78.36). Thus, it strongly implies that most of the DiCQAs are better absorbed than the PGAs.

## 1. Introduction

*Bidens pilosa* (BP) is an annual plant originating from South America and now found in almost all tropical and subtropical region countries around the World [1]. *Bidens pilosa* L. var. *radiata* Sch. Bip. Compositae (BPR), one of the subspecies of BP, is found in the wilds of Taiwan. The whole plant, including the root, stem, leaf and flower are used in various folk medicines and as a popular herbal tea ingredient. BPR has been proven to be effective for curing infectious hepatitis [2] and diabetes mellitus [3,4] when co-administerd with other medicines. Experiments with animal models have revealed BPR to possess promising anti-allergic [2], anti-ulcer [5] and anti-inflammatry bioactivities [6]. In a cell model, its ethanolic extract has shown excellent protective effect against oxidative damage in erythrocytes [7].

Approximately 200 different compounds have been discovered in BP and their structures have been well elucidated [1]. Among them, the main compounds including flavonoids and polyacetylenes have been separated and recognized as HPLC fingerprints of the bioactive compounds present in the extracts [8]. In addition, friedelanes and lupeol derivatives are the major terpenes present in BPR. Among the several categories of compounds investigated, the polyacetylenes, flavonoids, and their conjugated compounds are the most attractive [1]. In species *Bidens*, C_13_-phenylacetylenes and C_13_-acetylenes are the typical frequently found components and are taxonomically related. Usually, the former are more abundant [1]. Wat *et al.* indicated that the abundant occurrence of 1-phenylhepta-1,3,5-triyne in BPR leaves, stems, and roots is responsible for its intense UV absorptivity [9]. To date, twenty two kinds of polyacetylene glucoside (PAG)-related compounds have been found in *Bidens* species [10]. Among these, two compounds with highly potent antimalarial and antibacterial properties in *in vitro* and *in vivo* experiments have been investigated [11]. Another two active polyacetylenes exhibiting significant but differential ability to block endothelial cell proliferation, migration, and tube formation, while having only minimal or no detectable effect on cell proliferation of the tested cancer cell lines and immortalized keratinocytes also have been reported [12]. Attractively, PAGs happen to be the very common specific marker compounds of *Asteraceae* and *Campanulaceae* [13]. Another conspicuous class of compounds is represented by abundant amounts of caffeoylquinic acid (CQA) derivatives which are related to the antioxidant activity of BPR. These compounds with high antioxidant activities were also present in significant amounts in the leaves of sweet potato, being supposed to have potential for use as a functional food for improving human health [14]. Although diverse benefits of *B. pilosa* on human health have been investigated, there are still no studies pertaining to the bioavailability of the main components existing in *B*. *pilosa*, especially in BPR via intestinal transport mechanisms.

In this study, the optimum extraction conditions for the major bioactive components of BPR flowers were determined primarily by comparative analyses of total phenolics, total flavonoids and antioxidant activities. The extracts with the most antioxidant potential and their phytochemicals have been isolated and identified. Here, the identified six main compounds, including three DiCQAs and three PGAs were confirmed by using comparisons with authentic compounds in LC-MS analysis. Although these compounds have been identified in BPR [15,16], there have not been any reports of the intestinal absorption by using the human colonic cell line Caco-2. These six compounds exhibited differential transport in cells. In general, the DiCQAs are better absorbed than the PGAs in the flower extract. Moreover, PGA 5 (2β-d-glucopyranosyloxy-1-hydroxytrideca-5,7,9,11-tetrayne) showed the least absorption, with a significant efflux ratio when compared with the coefficient of apparent permeation. The dramatic differences in intestinal epithelial membrane transport between PGA 5 and the other five compounds are discussed.

## 2. Results and Discussion

### 2.1. Species Identification

Before the study, the identity of the plant was confirmed by Dr. Sy-Chian Liu (Department of Botany, National Chung-Hsing University, Taichung, Taiwan). Three voucher specimens were deposited at the Research Institute of Biotechnology, Hung-Kuang University, Taiwan. Moreover, the leaf samples were further used for the DNA extraction and the PCR amplification for the identification of species. From the sequence variation of internal transcribed spacer (ITS1 and ITS2), the sample species (*B. pilosa* var. *radiata*) used in the study was confirmed and obviously differentiated from the other two distributed species of *B. pilosa* var. *pilosa* and *B. pilosa* var. *minor* in Taiwan as compared to that of previous report [17].

### 2.2. Chemistry

#### 2.2.1. Yield, Total Phenolic and Flavonoid Contents of BPR Flower Extracts

**Figure 1:**
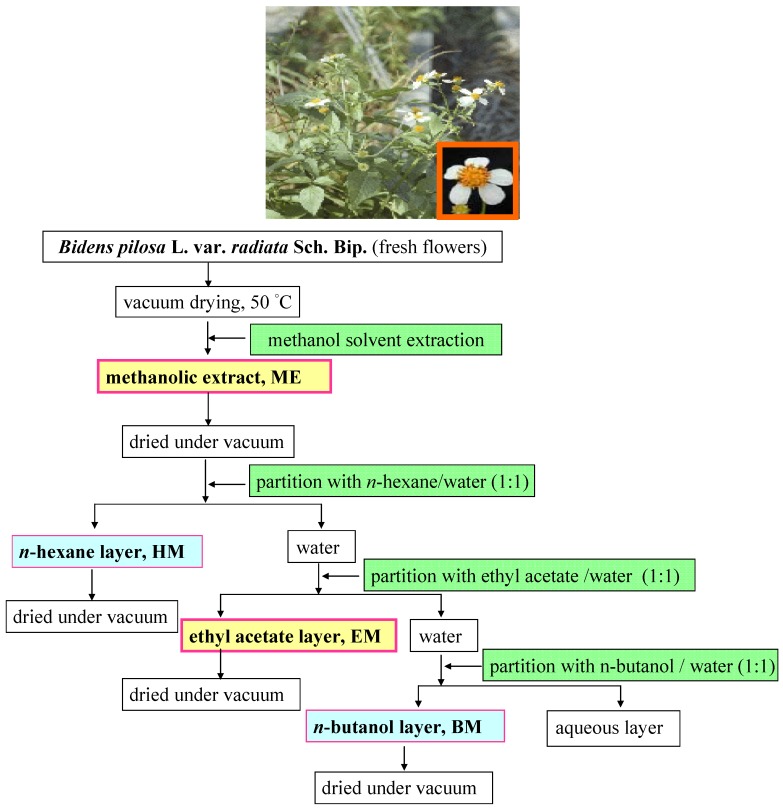
Flow chart of solvent extraction and liquid-liquid partition in different solvent polarity on flower part of *Bidens pilosa* L. *var. radiata*.

The extraction yield and the antioxidant activity of the extracts from plants highly depend on the solvent polarity, which determines both qualitatively and quantitatively the extracted antioxidant compounds [18]. Although water is a commonly used solvent in natural product research, previous reports indicate that methanol is recommended for the extraction of antioxidants in plants [18,19]. The use of methanol as the extraction solvent was performed at the beginning on the extraction of the bioactive compounds of BPR. The obtained extracts were then partitioned using three kinds of solvents of different polarities in order of nonpolar to polar including *n*-hexane, ethyl acetate and *n*-butanol for fractionating the compounds, as shown in the scheme of Figure 1. The yield was significantly (*p* < 0.05) higher with methanolic extraction (up to 40.40 ± 1.21%, w/w) than in the aqueous medium and the other solvents (Table 1). Both the antioxidant phenolics and flavonoids were also more abundant in the methanol extract (538.10 ± 0.96 and 235.06 ± 3.46 mg/g DW, respectively, Table 1). Thus, methanol extract was further applied in the partition to obtain the more effective antioxidants. The efficiency of different solvents for the partition of methanol extract (ME) of phenolics and flavonoids was found mostly to be in the order: EtOAc > *n*-butanol > *n*-hexane > water (Figure 1), except for the flavonoids in *n*-hexane fraction existed with a lower amount than that of aqueous fractione. The higher yields of phenolics and flavonoids should result in a better antioxidant capacity of the extract [20].

#### 2.2.2. Chemical Characterization by HPLC and LC-MS

**Figure 2:**
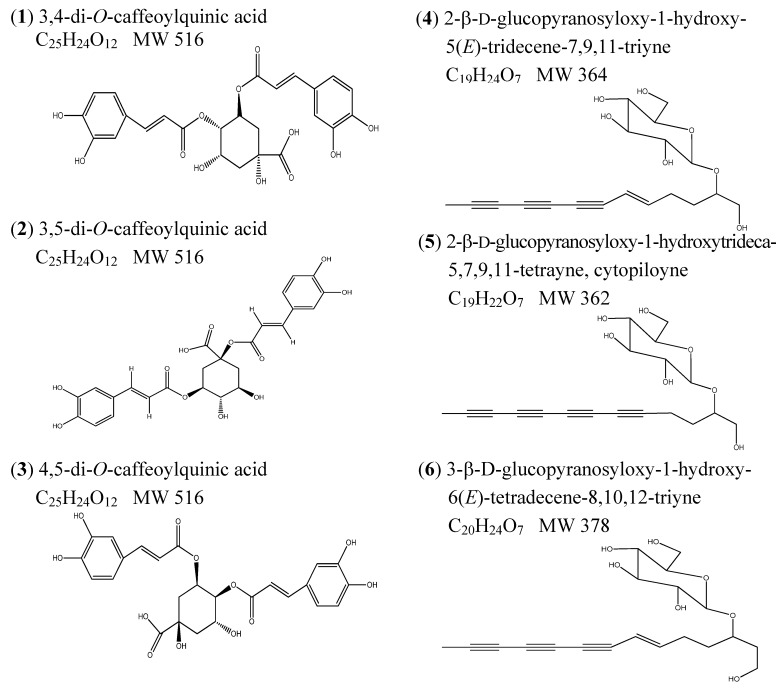
The six chemical structures identified from *Bidens pilosa* L. *var. radiata* flower extract.

This study was focused on the identification and quantification of di-*O*-caffeoylquinic acids (DiCQAs) and polyacetylene glucoside (PGA) compounds present in the different solvent extracts. Although HPLC-UV-MS coupled with an ESI interface has been used in the analysis of BPR extracts [16], we isolated and identify six authentic compounds (Figure 2) to apply in the identification of the compounds in BPR extract.

**Figure 3:**
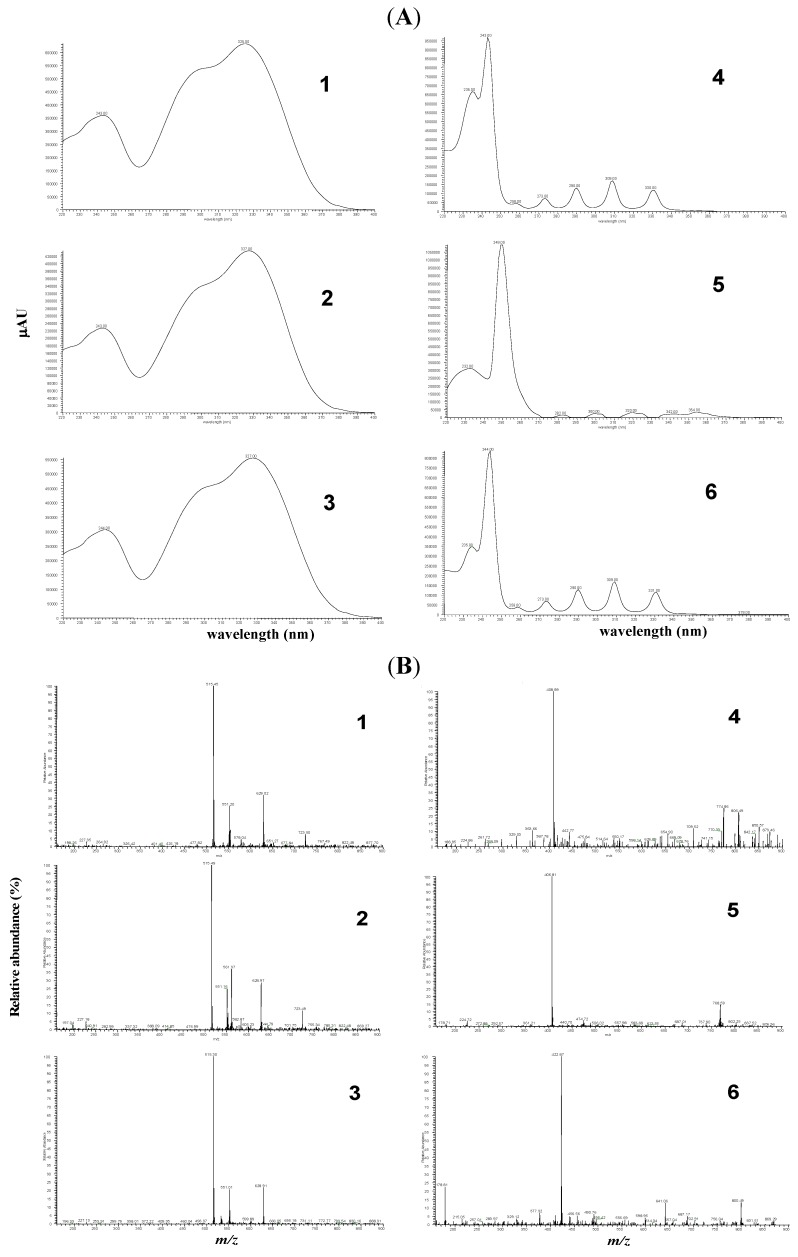
(**A**) UV Spectra (220–400 nm) of three caffeoylquinic acid derivatives (DiCQAs 1, 2 and 3) and three polyacetylenic glucosides (PGAs 4, 5 and 6) showing peaks with similar UV spectra, respectively; (**B**) The mass spectra obtained from a HPLC coupled to negative electrospray ionization (LC-ESI-MS) (DiCQAs 1, 2 and 3) and negative atmospheric pressure chemical ionization mass spectrometry (LC-APCI-MS) (PGAs 4, 5 and 6) based on *m/z* of molecular and product ions.

These compounds have been analyzed and characterized by using full spectrum UV-vis and their MS spectrum, as shown in Figure 3A,B, in the identification of the major di-CQAs and PGAs. Three di-CQAs compounds (3,4-, 3,5- and 4,5-di-*O*-caffeoylquinic acids, respectively, Figure 3A) showed a similar UV spectrum that was characteristic of flavonoid compounds [21] and presented the same molecular ion at *m/z* 515 [M−H]^−^ (Figure 3B). The other three PGAs (PGA 4: 2-β-d-glucopyranosyloxy-1-hydroxy-5(*E*)-tridecene-7,9,11-triyne, PGA 5: 2-β-d-glucopyranosyloxy-1-hydroxytrideca-5,7,9,11-tetrayne and PGA 6: 3-β-d-glucopyranosyloxy-1-hydroxy-6(*E*)-tetradecene-8,10,12-triyne) also presented a similar result.

**Figure 4:**
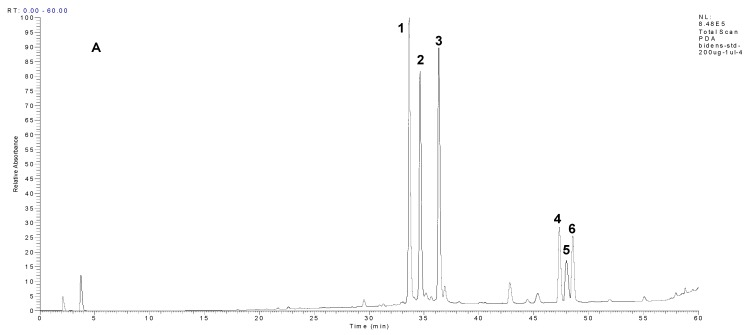

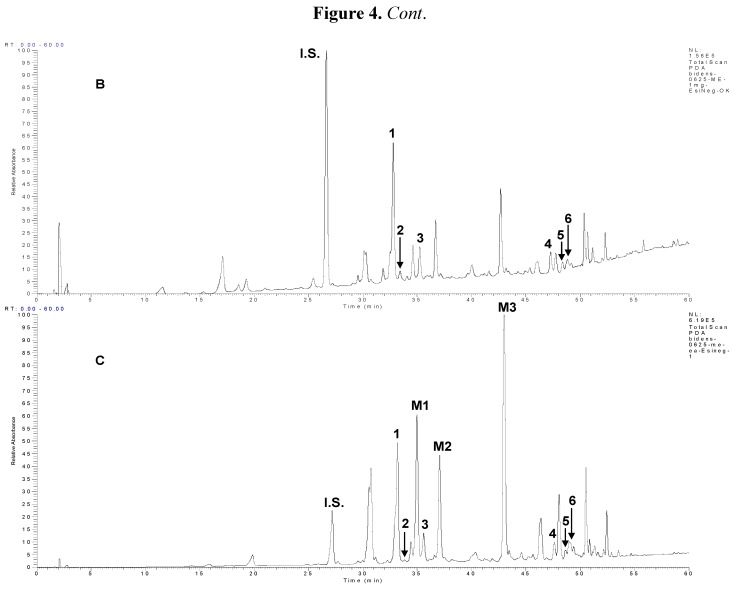
Representative HPLC chromatograms: (**A**) the purified six authentic compounds; (**B**) aqueous extract, crude methanol extract and its different solvent partition fractions (1 mg/mL) of flower part of BPR. AE: aqueous extract, ME: methanol extract, HM: *n*-hexane fraction, EM: ethyl acetate fraction, BM: *n*-butanol fraction, AM: the remaining aqueous fraction. The peak number and its chemical structure are referred to Figure 2. Characteristic UV spectra, negative- ESI and APCI mass spectra are referred to Figure 3A,B, respectively.

We found that polyacetylenic compounds have typical absorption bands in their UV spectra (maximums at 243 and four intense absorption bands at 273, 290, 309 and 331 nm in a similar absorption coefficient) that are attributed to the carbon-carbon triple bonds. They are easily identified in extracts by DAD owing to the very characteristic UV-spectra resulting from their conjugated triple bonds [22]. However, the UV and MS data were insufficient for on-line differentiation between these compounds and the identification confirmed by matching the retention time against those of standard compounds would be necessarily. The authentic compounds used for the identification on these compounds were obtained by preparative HPLC fractionation according to a previous report [23]. To determine the content of extract, HPLC analysis with the calibration curves of six authentic compounds combined the internal standard 2,4,5-trimethoxybenzoic acid was carried out (Figure 4A) for the quantification of compounds in the prepared extract (ME) and fractions (EA/ME) as shown in Figure 4B,C. DiCQA and PGA determinations in methanolic extract (ME) and its fraction (EM) obtained from the solvent-solvent partition with ethyl acetate were performed at 220–400 nm full scan and the 325 and 245 nm wavelengths were selected to facilitate the identification of the different DiCQA and PGA families present in ME and EM (data not shown). HPLC-DAD profiling (Figure 4B,C) revealed the similar major peaks as well as several minor peaks for the ME and EM extracts. However, there were three compounds highlighted as M1, M2 and M3, respectively, that were significantly enhanced after the fractionation by ethyl acetate as shown in Figure 2C. They were tentatively identified as luteolin-4-*O*-glucoside, luteolin-7-*O*-glucoside and luteolin, respectively by LC-MS analysis and comparison to previous reports [1,24].

The concentrations of identified components were quantified by using the internal standard method obtained from the standard curves of the six authentic compounds covering the concentration ranges of 20–200 μg/mL (r^2^ = 9978–9996). Results are listed in Table 2.

According to ANOVA, significant differences of the content in each compound were observed between the studied extracts before and after fractionation with ethyl acetate. The most abundant component was 3,5-DiCQA (220 ± 0.13 mg/g of DW) followed by PGA 4 (109.0 ± 0.99 mg/g of DW) in the ethyl acetate fraction. Compared to previous report of Chiang *et al.* [16], the ethyl acetate fractionation resulted in a more abundant and simple composition than *n*-butanol fractionation.

#### 2.2.3. Free Radical Scavenging Activity of Different Solvent Extracts

The free radical 2,2’-diphenyl-1-picrylhydrazyl (DPPH) scavenging activity was determined spectrophotometrically by measuring the disappearance of the radical at 515 nm, which has been widely used in the evaluation of the antioxidant activity of natural products [25,26,27]. As shown in Table 3, influence of solvent polarity on extraction and partition on the free DPPH scavenging activity might have indeed existed, whereby the EM presented the highest scavenging activity, better than all the other extracts or solvent solvent fractions of ME and the most approximate activity to Trolox (a potent antioxidant used as a positive control). There was a correlation between scavenging activity and total phenolic content as well as total flavonoid content. The further comparisons of activity were expressed as the IC_50_ values as listed in Table 3, which clearly indicated the minimum concentration for inhibition of 50% DPPH radical derived from EM is 82.8 ± 3.7 μg/mL.

#### 2.2.4. Trolox Equivalent Antioxidant Capacity (TEAC)

The TEAC assay is based on the inhibition of the absorbance of radical cations of 2,2’-azino-bis-3-ethylbenzthiazoline-6-sulphonic acid (ABTS) by antioxidants when ABTS is incubated with a peroxidase and H_2_O_2_ [28]. Among the six extracts, EM showed the highest antioxidant capacity, followed by ME and HM extracts. BM and AE showed a moderate activity, while the AM was the lowest (Table 3). The IC_50_ values (Table 3) also clearly showed that EM presented the highest antioxidant activity with the lowest concentration at 135 ± 1.01 µg/mL. The better antioxidant activity might be related to the compositions of the extract, especially the most abundant content of 3,5-DiCQA, which has been reported to possess very strong ABTS^●+^ radical cation scavenging capability with IC_50_ value at 10 μM [29]. There was an intense correlation of antioxidant activity between the results of ABTS assay and DPPH assay in the study with a similar trend in the prepared extracts. However, the ABTS assay has been indicated to better reflect the antioxidant contents in a variety of foods than the DPPH assay [30].

#### 2.2.5. Ferric Reducing Antioxidant Power (FRAP)

**Figure 5:**
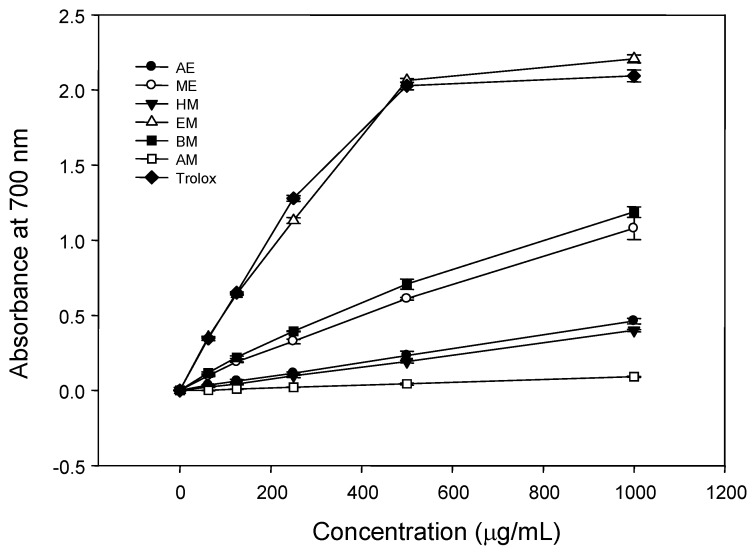
Reducing powers of different *Bidens pilosa* flower extracts. Trolox was used as positive control. Each bar represents the means ± SD of triplicate experiments. AE: aqueous extract, ME: methanol extract, HM: *n*-hexane fraction, EM: ethyl acetate fraction, BM: *n*-butanol fraction, AM: the remaining aqueous fraction.

The FRAP assay is different from the aforementioned methods as there are no free radicals involved but rather the reduction of Fe^3+^ to Fe^2+^ is determined. Since reducing activity of a compound serves as a significant indicator of its antioxidant capability [31], the different BPR extracts were assayed for their reducing power. The OD at 700 nm increased in a dose responsive manner from 0 to 1,000 µg/mL (Figure 5). Trolox was also used as a positive standard. As shown in Figure 5, the EM extract showed with the highest reducing powder, even better than Trolox, at the dosages of 200–1000 µg/mL. Thus, the results indicated that EM is a rich source of antioxidants with specific compounds and profiles. Further studies on the bioavailability of the extracts would be necessarily to the better understand the potential use of BPR.

### 2.3. Permeability and Efflux Ratio in Caco-2 cells

**Figure 6:**
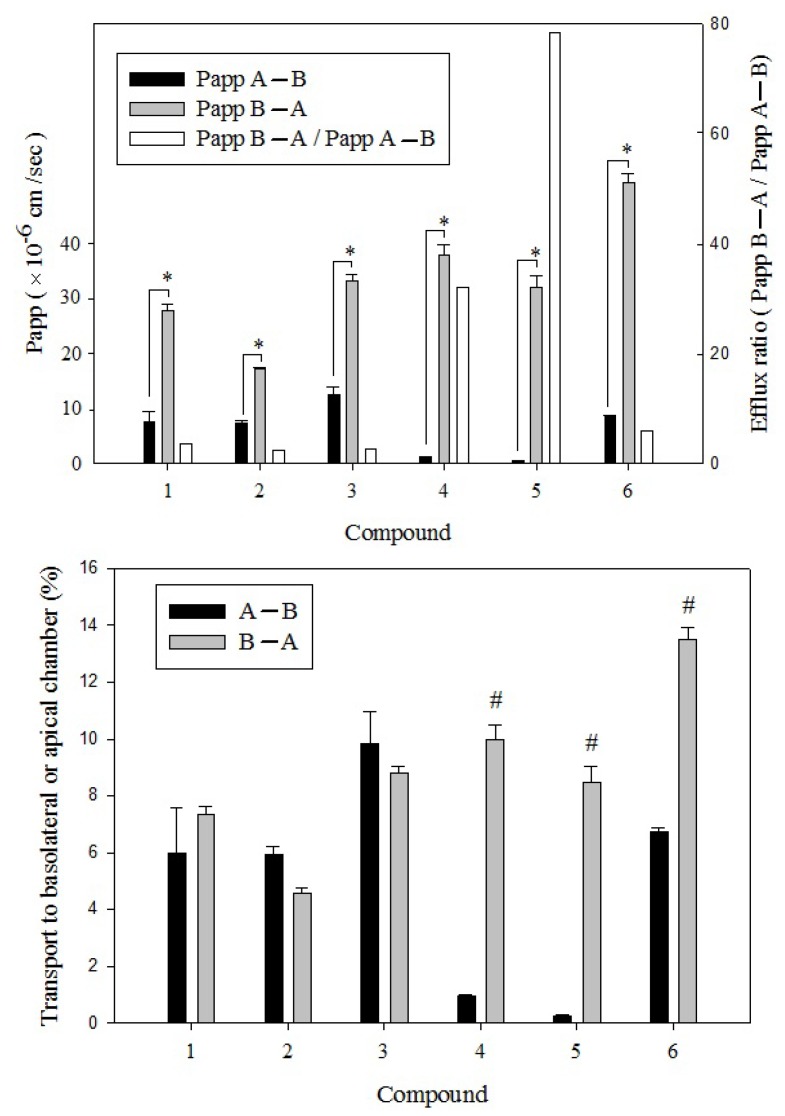
The apparent permeability coefficient (Papp) (upper panel) and the transport percentage (lower panel) of each compound in EM of BPR in the secretory (**B**–**A**) direction (□) and in the absorptive (**A**–**B**) direction (⬛), and the efflux ratio (□). The structures and names of compounds 1–6 are referred to Figure 2. ^*^ Papp value of each tested compound in the secretory (**B**–**A**) direction significantly greater compared with Papp value in the absorptive (**A**–**B**) direction (*p* < 0.05). ^#^ Transport of each compound in EM of BPR in the secretory (**B**–**A**) direction significantly greater compared with in the absorptive (**A**–**B**) direction (*p* < 0.05).

To investigate the intestinal absorption and transport properties of the three DiCQAs and three PGAs, 14-day cultured Caco-2 monolayer cells were used for the bidirectional transport study. As studied before, Qiang *et al.* [32] indicated that the compounds in herbal extracts have similar uptakes as those found using pure compounds. Our hypotheses were that the absorbability of the aforementioned major six compounds in EM extract of BPR would be independent of the extract matrix. Before the study, the integrity of cell monolayers presented at the TEER values ≥ 350 Ω/cm^2^ were determined and considered acceptable for this study [33]. Owing to the incubations of tested samples for up to 60 min were acceptable and resulted in a linear transport, we conducted all of the comparative experiments within 1-h incubation. As shown in Figure 6, the Papp for the apical to basolateral flux of Di-CQAs 1, 2 and 3, and PGAs 4 and 6 were higher than the paracellular transport marker mannitol (0.43 ± 0.10 × 10^−6^ cm/s) with an exception of PGA 5, which was lower than the Papp for mannitol in the same inserts. In contrast, the Papp for the basolateral to apical flux of PGA 5 was closed to that of propranolol (39.4 ± 4.12 × 10^−6^ cm/s, data not shown), a passive transcellular transport marker. PGA 5, with four carbon-carbon triple bonds in the structure, was distributed extremely rapidly from the apical solution into the cell membranes within 60 min.

The more carbon-carbon triple bonds in the structure resulted in higher lipophilicity. Thus, the compatibility between the molecules and cell membranes would be stronger. In the quantitative analysis of PGA 4, a molecular structure similar to PGA 5 with the same glycosidic bond linked position at C-2, but with one less triple bond, was observed to have a lower relative Papp than that of PGA 5 (Figure 6). In addition, a lesser efflux ratio of PGA 4 (32.1) and PGA 6 (6.03) were presented with faster basolateral to apical transport than in the apical to basolateral direction across the Caco-2 cell monolayer. In general, a value of <2.0 for efflux ratio implies that only passive diffusion is involved in drug transport, whereas efflux ratio values that greatly exceed 2.0 suggest that test compounds are substrates of efflux transporters at the apical membrane [34].

As mentioned, the difference of structure between the glycosidic linkage position and the carbon-carbon triple bonds number was supposed to be a highly possible influencing factor. PGAs 4 and 6 characterized with the same alkyne and alkene linkages only the position difference of the glycosidic bond between C-2 and C-3 are quite different from the structure of PGA 5 (Figure 6). Thus, an efflux pump localized on the apical membrane, with a higher specificity for PGA 5 than the other PGAs was supposed to be responsible for these observations [35]. However, PGA 5 has been found to be the most effective compound for increasing serum insulin, reducing blood glucose levels, and for reducing glycosylated HbA1c levels in db/db mice experiments compared to the other two polyacetylenic glucosides when administered i.p. [14]. The conflicting findings were likely due to the higher efficiencies of metabolites from PGA than the original form or the interactive effects occurred between the compounds of extract, which will be the subject of further investigation.

## 3. Experimental 

### 3.1. Chemicals and Reagents

2,2′-Azino-bis(3-ethyl-benzthiazoline-6-sulfonic acid) (ABTS), butylated hydroxytoluene (BHT), ferric chloride, gallic acid, quercetin, dexamethasone, potassium ferricyanide (K_3_Fe(CN)_6_), insulin, pyrogallol, propidium iodide (PI), Folin-Ciocalteau phenol reagent, propranolol hydrochloride, 3-isobutyl-1-methylxanthine, trypan blue, 3-(4,5-dimethylthiazol-2-yl)-2,5-diphenyltetrazolium bromide (MTT) and 2,4,5-trimethoxybenzoic acid (TMBA) were provided by Sigma-Aldrich (St. Louis, MO, USA). Protein assay kits were supplied by Bio-Rad Biotech (Hercules, CA, USA). Acetonitrile, methanol, ethyl acetate, and dimethylsulfoxide (DMSO) were purchased from Merck Co. (Darmstadt, Germany). Penicillin-streptomycin solution (10,000 U/mL penicillin and 10,000 μg/mL) was from HyClone (Logan, UT, USA). The six purified and identified phytochemicals from BPR including (1) 3,4-di-*O*-caffeoylquinic acid; (2) 3,5-di-*O*-caffeoylquinic acid; (3) 4,5-di-*O*-caffeoylquinic acid; (4) 2-β-d-glucopyranosyloxy-1-hydroxy-5(*E*)-tridecene-7,9,11-triyne; (5) 2-β-d-glucopyranosyloxy-1-hydroxytrideca-5,7,9,11-tetrayne, cytopiloyne and (6) 3-β-d-glucopyranosyloxy-1-hydroxy-6(*E*)-tetradecene-8,10,12-triynewere kindly donated by Aggie Bionatural Technology (Taipei, Taiwan).

### 3.2. Source of Bidens pilosa L. var. radiata Sch. Bip. (BPR)

Fresh flower samples were harvested during flowering season in August 2008 from the middle Taiwan area. Before the study, the variety of botanical specimens was identified according to previous report [17] to confirm the exact species. On harvesting, samples were immediately desiccated at 50 °C in a vacuum oven and were ground to a fine powder (20 mesh) in a comminuted mill (Retsch Ultra Centrifugal Mill and Sieving Machine, Type ZMI, Haan, Germany). The powder was stored in dark at −80 °C until use.

### 3.3. Solvent Extraction and Partition Fractionation

Thirty grams of finely milled freeze-dried flowers were separately extracted with deionized water and methanol, each using 600 mL of the solvent (3:60). The condition was performed in triplicate. Sample vials were placed in a sonicator bath (Delta Sonicator DC200H, LMI Co. Ltd., Taipei, Taiwan) at ambient temperature for 60 min. The more effective methanolic extract (ME) was further submitted to liquid-liquid partition sequentially using the n-hexane, ethyl acetate, *n*-butanol leaving the aqueous phase. At the beginning of fractionation, the desiccated ME was redissolved in saturated brine (200 mL) which was then subjected to solvent-solvent partition (×3) with *n*-hexane (1:1, v/v). The remaining aqueous layer was further treated with ethyl acetate at a 1:1 (v/v) ratio of ethyl acetate/water. The extraction was repeated three times. Again, the remaining aqueous layer was further treated with *n*-butanol in a 1:1 (v/v) ratio of *n*-butanol/water. The same extracts were combined, filtered, vacuum concentrated and then lyophilized to yield AE and ME, and the *n*-hexane fraction from the ME (HM), the ethyl acetate partition from the ME (EM), the *n*-butanol fraction from the ME (BM) and finally the remaining aqueous extract (AM), respectively. Figure 1 briefly outlines the preparation of methanol extract and four fractions of dried flowers of *B. pilosa*. The crude extracts and fractions were evaluated for their total phenolics, total flavonoids and antioxidant activities. Methanolic extract (ME) and EM partition fraction were subsequently reconstituted in dimethyl sulfoxide prior to evaluation of the transport of Caco-2 cell line.

### 3.4. Assay of Total Phenolics

The method of Silvia Taga *et al.* [36] was followed for determination of total phenolics. Briefly, extract of lyophilized BPR (1 mg) was carefully weighed and dissolved in methanol (1 mL). To an aliquot (0.1 mL), Na_2_CO_3_ (2%, 2 mL) was added, mixed well, and left to stand for 2 min. To the mixture Folin-Ciocalteau reagent (0.1 mL, 50%) was added. The mixture was left to stand for 30 min and the optical density was taken at 750 nm with a spectrophotometer (Thermo Biomate 5, Thermo Electron Corporation, San Jose, CA, USA). A calibration curve was similarly established using authentic gallic acid. The amount of phenolics was expressed as mg gallic acid equivalent per g of extract (mg GE/g).

### 3.5. Assay of Total Flavonoids

The assay for total flavonoids was carried out according to the method of Zhishen *et al.* [37]. In brief, to sample solution (250 μL, 5 mg/mL), double distilled water (1.25 mL) and NaNO_2_ solution (75 μL, 5% w/v) were added and mixed well. On standing for 6 min, AlCl_3_**·**6H_2_O (150 μL, 10% w/v) was added and mixed well. After 5 min, 1 M NaOH (0.5 mL) and water was added to make up a total volume of 2.5 mL. After mixing well, the optical density was read at 510 nm. A calibration curve was similarly established using authentic quercetin. The amount of isoflavonoids was expressed as mg quercetin equivalent per g of extract (mg QE/g).

### 3.6. Evaluation of Antioxidant Activity

#### 3.6.1. Free Radical Scavenging Activity Determination (DPPH)

The stable 1,1-diphenyl-2-picrylhydrazyl radical (DPPH) was used for determination of free radical-scavenging activity of the extracts [38]. Different concentrations of each extract and fraction were added, at an equal volume (0.1 mL), to methanolic solution of DPPH (0.5 mM). After 30 min at room temperature, the absorbance was recorded at 517 nm. The experiment was repeated for three times. IC_50_ values denote the concentration of sample, which is required to scavenge 50% of DPPH free radicals.

#### 3.6.2. Trolox Equivalent Antioxidant Capacity (TEAC) 

For TEAC (or the more specified definition as ABTS radical cation scavenging activity) evaluation, a slightly modified method of Arnao *et al.* [39] was followed. The stable ABTS^∙+^ reagent is generated by mixing with final concentrations of 0.1 mM ABTS, 4.4 unit/mL horseradish peroxidase (Sigma-Aldrich) and 50 μM H_2_O_2_ in 50 mM phosphate buffer (pH 7.4) and allowing to stand in the dark at room temperature overnight to form a blue-green color solution. Each of the extract or partition (50 μL) was added into the solution, the decrease of the absorbance at 734 nm was measured after 10 min. The results were expressed as IC_50_ values with the concentration (in μg/mL) of sample required for 50% inhibition of ABTS^●+^.

#### 3.6.3. Ferric Reducing Antioxidant Power Determination

The reducing power was determined according to the method of Oyaizu [40]. Various concentrations of each extract and partition (0.2 mL) were mixed with sodium phosphate buffer (0.2 mL, 0.2 M, pH 6.6) and 1% potassium ferricyanide (0.2 mL). The mixture was incubated at 50 °C for 20 min. After 10% trichloroacetic acid (w/v) (0.2 mL) were added, the mixture was centrifuged at 6,000 *g* for 10 min. Supernatant obtained hereafter (100 μL) was immediately mixed with distilled water (100 μL) and 0.1% of ferric chloride (20 μL). The absorbance was measured after 10 min at 700 nm. Higher absorbance value indicates higher reducing power. Trolox was used a positive control.

### 3.7. HPLC and LC-MS Analysis of Main Compounds in BPR

#### 3.7.1. Preparation of Standard Stock and Sample Solutions

Each authentic constituent was dissolved in 60% ethanol to obtain different strengths of 10, 20, 50, and 100 μg/mL after spiking with internal standard solution (2,4,5-trimethoxybenzoic acid, TMBA, 25 μg/mL). The solutions were made up with methanol to a final volume of 1 mL and filtered through a nylon syringe filter (0.22 μm, 13 mm diameter) for HPLC analysis. The stock solution of internal standard was prepared by dissolving TMBA in methanol to make a concentration of 1 mg/mL and filtered through a nylon membrane disc filter (0.22 μm, d = 13 mm). To aliquots of 5 mg of aqueous-, methanol-, *n*-hexane-, ethyl acetate- and *n*-butanol solvent extract were separately made up to a final volume of 10 mL with methanol. A suitable amount of internal standard (TMBA) was added to give a final concentration of 2.5 μg/mL before HPLC analysis. 

#### 3.7.2. HPLC Operation

The HPLC analysis was carried out by using a Hitachi L2130 HPLC. The column (ℓ × i.d. = 4.6 mm × 250 mm) was packed with Phenomenex Luna C18 5 μm. A Hitachi L2400 UV detector was installed and monitored at 240 nm. An aliquot of sample solution (20 μL) was injected and the separation was conducted by eluting with a gradient mobile phase controlled at a flow rate 1 mL/min. The gradient elution solvent was made up of two solvents. Mobile phase A was an aqueous phase containing 0.05% trifluoroacetic acid. Mobile phase B was acetonitrile containing 0.05% trifluoroacetic acid. The entire course of programmed gradient elution was as follows: from 0–40 min, solvent A:B = 95:5, from 40–65 min, by solvent A:B = 70:30, from 65–70 min, with solvent A:B = 60:40, from 70–75 min, in solvent A:B = 5:95, from 75–80 min, by solvent A:B = 5:95, and from 80 min on, by solvent A:B = 95:5.

#### 3.7.3. LC-MS Analysis

The HPLC Finnigan Surveyor module separation system connected with a precolumn [SecurityGuard C18(ODS) 4 mm × 3.0 mm ID, Phenomenex Inc., Torrance, CA, USA] and an analytical column [150 × 2.0 mmi.d., 3 μm Luna C18 (2), Phenomenex] was used for LC-MS analysis. The elution mobile system comprised two solvents, solvent A—aqueous phase containing 0.1% formic acid, and solvent B—acetonitrile containing 0.1% formic acid. The entire course of programmed gradient elution was carried out as follows: 0–3 min, isocratic with 5% B; 3–20 min, with 5%–20% B; 20–35 min, with 20%–30% B; 35–50 min, with 30%–40% B, 50–55 min, with 40%–95% B, followed by 5 min of isocratic 95% B and returning to initial conditions in 10 min. The HPLC was operated at a flow rate of 0.2 mL/min. A photodiode array detector (PAD, Thermo Electron Co., Waltham, MA, USA) recording at 240, 280, and 425 nm and scanning from 200 to 600 nm was interfaced with the Finnigan LCQ Advantage MAX ion trap mass spectrometer with an electrospray ionization (ESI) source and operated in the negative model. The operation conditions were: spray needle voltage, 4.0 kV; tube lens offset, −35 V; ion transfer capillary temperature, 280 °C; nitrogen sheath gas, 45; and auxiliary gas, 5 (arbitrary units). All the data was processed with the Xcalibur 2.0 data system (Thermo Electron Co.). The identification of compounds was carried out comparing their retention times, UV-Vis information and mass spectra provided by ESI-MS and ESI-MS/MS with those of authentic standards. In the polyacetylenes analysis, the MS analysis showed that polyacetylenes are non-detectable among the protonated molecular ions [M−H]^−^. If the ionization result was unavailable in ESI, the atmospheric pressure chemical ionization (APCI) in negative mode was used in accordance with the previous study [41]. All data were acquired in the full-scan negative mode from *m*/*z* 150 to 900. Capillary temperature was 200 °C, APCI vaporizer temperature 400 °C, nitrogen sheath gas 50, auxiliary gas 10 (arbitrary units), source current 5.0 μA and source voltage 4.5 kV.

### 3.8. Biological Evaluation 

#### 3.8.1. Cell Culture

The human intestinal epithelial Caco-2 cell line (BCRC 60182) purchased from the Bioresource Collection and Research Center (BCRC) in Taiwan, was applied for evaluating the bioavailability of components in the most antioxidant potential candidate from EA/ME extract of BPR. It was cultured in Dulbecco’s Modified Eagle Medium (DMEM medium, Hyclone, Logan, UT, USA) containing high glucose (4.5 g/L) plus 10% fetal bovine serum (Biological Industries, Beit Haemek, Israel), 1% l-glutamine and 1% penicillin-streptomycin (Hyclone) at 37 °C, 5% CO_2_ with constant humidity controlled environment. The medium was replaced three times a week during cell growth and differentiation. Cells used in this study were between passages 25 and 40.

#### 3.8.2. Transepithelial Transport and Uptake 

After the cells in the flask grew to 90%–100% confluency, cells were trypsinized and seeded on 12 mm transwells with 0.4 µm pore polyester membrane insert (product #3460, Corning Inc., Corning, NY, USA) at a density 2 × 10^5^ cells/mL. Culture medium (0.5 mL and 1.5 mL, respectively) was added into the space between the transwell and the plate. The culture was incubated at 37 °C under 5% CO_2_ atmosphere and the medium was changed fresh every 48 h. The transepithelial electrical resistance (TEER) was measured with Millicell-ERS epithelial Voltohmmeter (Millipore, Bedford, MA, USA), which was reflected to the tightness of intercellular junctions and only cells with TEER (in Ω/cm^2^) >300 Ω/cm^2^ (at 14 day) were used for permeability study [42].

Before conducting the permeation test, the trasnswell insert was emerged and rinsed in 25 mM HEPES (pH 7.4 Hank’s Balanced Salt Solution, HBSS) and rinsed twice at 37 °C under 5% CO_2_ atmosphere, each time for 30 min. The paracellular marker molecule mannitol (low permeability), propranolol (highly permeability), and EM fraction (1 mg/mL) were separately dissolved in DMSO and diluted with HBSS to final concentrations of 50 μM, 10 μM, and 1 mg/mL (at non-cytotoxic concentration), respectively. The solutions were respectively transferred into the apical and basolateral zones of the transwell insert with the other side filled up with HBSS. The same pH (7.4) was used both in apical-to-basolateral (A→B) and basolateral-to-apical (B→A) transport directions during each experiment. The whole apparatus was incubated at 37 °C under 5% CO_2_ atmosphere and the permeation was examined at time point 5, 15, 30, and 60 min, respectively. Permeates obtained from various direction were analyzed with HPLC and LC-MS according to the aforementioned method. The apparent permeability coefficients (P_app,_ cm/s) and the efflux ratio were calculated according to the literature [43]:

P_app_ = (V/A × C_0_) × (dQ/dt)

where V is the volume of receiver part (0.5 mL and/or 1.5 mL).

A is the surface area of the cell monolayer (1.1 cm^2^).

C_0_ is the initial concentration of the extract (1 mg/mL).

dQ/dt is the cumulative transport rate (mg/mL/min).

Efflux ratio = P_app,B__→A_/P_app,A__→B_

### 3.9. Statistical Analysis

Results were expressed in mean ± SD. Data was analyzed with unpaired Student’s *t*-test to assess the group difference in sample and dosage. Differences of each compound within the two extracts were compared by analysis of variance (ANOVA). A level with *p* < 0.05 was considered to be significant.

## 4. Conclusions 

The results of this study clearly show that ethyl acetate soluble fraction of the methanolic extract has a significant effect on the yield, total phenolic and total flavonoid contents, and antioxidant activities of BPR. In addition, the *in vitro* bioavailability of extract in a cell model showed that the better absorbtion of DiCQAs than from the PGAs was demonstrated in the major two classes of components of the BPR extract. These findings suggest the potential use of BPR extract (including DiCQAs and PGAs), rather than PGAs alone, for treating diabetes mellitus.

## Figures and Tables

**Table 1:** Yield, total phenolic contents and total flavonoids in different solvent extracts of *Bidens pilosa* L. *var. radiata* flower.

Extract ^1^	Yield (%)	Content (mg/g dry wt.)	
		Total phenolics ^2^	Total flavonoids ^3^
AE ME	31.05 ± 10.75 ^b^ 40.40 ± 1.21 ^a^	105.05 ± 3.73 ^d^ 538.10 ± 0.96 ^b^	172.11 ± 2.14 ^d^ 235.06 ± 3.46 ^c^
HM	3.83 ± 0.50 ^d^	51.71 ± 4.76 ^e^	79.82 ± 2.45 ^f^
EM	5.97 ± 0.35 ^d^	752.15 ± 7.53 ^a^	469.10 ± 0.97 ^a^
BM AM	5.56 ± 1.65 ^d^ 15.61 ± 0.14 ^c^	236.02 ± 1.18 ^c^ 25.12 ± 0.43 ^f^	363.19 ± 5.07 ^b^ 108.08 ± 8.49 ^e^

^1^ AE: aqueous extract; ME: methanol extract; HM: *n*-hexane fraction, EM: ethyl acetate fraction, BM: *n*-butanol fraction; AM: the remaining aqueous fraction; ^2^ mg gallic acid equivalent /g extract weight; ^3^ mg quercetin equivalent /g extract weight. Each value represents the means ± SD of triplicate experiments. Columns with a different letter are significantly different (*p* < 0.05).

**Table 2:** Content of the six major components from methanolic extract and its ethyl acetate fractionate of *Bidens pilosa* flower.

Peak No. ^1^	Retention Time (min)	Content (mg/g dry weight) ^2^	
		Methanolic extract	Ethyl acetate fractionate
1	33.72	33.4 ± 0.01 ^b3^	39.8 ± 0.32 ^a^
2	35.15	123.0 ± 0.55 ^b^	220.0 ± 0.13 ^a^
3	36.95	58.3 ± 0.03 ^b^	67.3 ± 0.87 ^a^
4	49.12	76.9 ± 0.41 ^b^	109.0 ± 0.99 ^a^
5	49.68	16.4 ± 0.09 ^b^	23.0 ± 0.29 ^a^
6	50.27	15.9 ± 0.07 ^b^	25.8 ± 0.56 ^a^

^1^ The identified compound name and structure were referred to Figure 2; ^2^ Each value in the determined value represents a mean ± SD (n = 3); ^3^ Mean values followed by different letters are significantly different from each other in the same raw at *p* < 0.05 level.

**Table 3:** Scavenging effects of different solvent extracts from *Bidens pilosa* on the DPPH radical and TEAC.

Extract ^1^	DPPH IC_50_ (μg/mL) ^2^	TEAC IC_50_ (μg/mL) ^3^
AE	1045.4 ± 63.6 ^b^	818.5 ± 74.3 ^b^
ME	207.3 ± 43.5 ^d^	340.3± 29.5 ^c^
HM	260.2 ± 5.0 ^cd^	294.4 ± 18.0 ^cd^
EM	82.8. ± 3.7 ^e^	135.3 ± 1.0 ^d^
BM	284.4 ± 5.1 ^c^	365.3 ± 13.5 ^c^
AM	1634.2 ± 48.4 ^a^	1633.4 ± 224.2 ^a^

^1^ AE: Aqueous extract, ME: Methanol extract. The extract obtained by using liquid-liquid partition on methanolic extract was extracted sequentially by using *n*-hexane, ethyl acetate, *n*-butanol and the remaining aqueous part. They were denoted as HM, EM, BM and AM, respectively. Each value represents the means ± SD of triplicate experiments; ^2,3^ IC_50_: concentration (in mg/mL) necessary for inhibition 50% DPPH radical and ABTS^●+^ cation radical, respectively. Columns with a different letter are significantly different (*p* < 0.05).

## References

[1] Silva L.F., Fischer D.C.H., Tavares J.F., Silva M.S., de Athayde-Filho P.F., Barbosa-Filho J.M. (2008). Compilation of secondary metabolites from *Bidens pilosa* L.. Molecules.

[2] Horiuchi M., Seyama Y. (2008). Improvement of the antiinflamatory and antiallergic activity of *Bidens pilosa* L. var. *radiata Scherff* treated with enzyme (cellulosine). J. Health Sci..

[3] Hsu Y.J., Lee T.H., Chang C.L.T., Huang Y.T., Yang W.C. (2009). Anti-diabetic mechanism of *Bidens pilosa* extract: Stimulation of insulin release from beta cells. J. Ethnophamacol..

[4] Chang S.L., Chang C.L.T., Chiang Y.M., Hsieh R.H., Tzeng C.R., Wu T.K., Sytwu H.K., Yang N.S., Shyur L.F., Yang W.C. (2004). Polyacetylenic compound and butanol fraction from *Bidens pilosa* Linn can modulate the differentiation of helper T cells and prevent autoimmune diabetes in non-obese diabetic mice. Planta Med..

[5] Alvarez A., Pomar F., Sevilla M.A., Montero M.J. (1999). Gastric antisecretory and antiulcer activities of an ethanolic extract of *Bidens pilosa* L. var. radiata Schult. Bip. J. Ethnopharmacol..

[6] Pereira R.L.C., Ibrahim T., Lucchetti L., Silva A.J.R., Moraes V.L.G. (1999). Immunosuppressive and anti-inflammatory effects of methanolic extract and the polyacetylene isolated from *Bidens pilosa* L.. Immunopharmacology.

[7] Bairwa K., Kumar R., Sharma R.J., Roy R.K. (2010). An updated review on *Bidens Pilosa* L.. Pharma. Chem..

[8] Cortés-Rojas D.F., Chagas-Paula D.A., da Costa F.B., Souza C.R.F., Oliveira W.P. (2012). Bioactive compounds in *Bidens pilosa* L. populations: A key step in the standardization of phytopharmaceutical preparations. Rev. Bras. Farmacogn..

[9] Wat C.K., Johns T., Towers G.H.N. (1980). Phototoxic and antibiotic activities of plants of the Asteraceae used in folk medicine. J. Ethnopharmacol..

[10] Ganjewalaa D., Kumara S., Ambikaa K., Luthrab R. (2008). Plant polyacetylenic glycosides occurrence, biosynthesis and biological activities. Pharmacologyonline.

[11] Tobinaga S., Sharma M.K., Aalbersberg W.G.L., Watanabe K., Iguchi K., Narui K., Sasatsu M., Waki S. (2009). Isolation and identification of a potent antimalarial and antibacterial polyacetylene from *Bidens pilosa*. Planta Med..

[12] Wu L.W., Chiang Y.M., Chuang H.C., Wang S.Y., Yang G.W., Chen Y.H., Lai L.Y., Shyur L.F. (2004). Polyacetylenes function as anti-angiogenic agents. Pharm. Res..

[13] Minto R.E., Blacklock B.J. (2008). Biosynthesis and function of polyacetylenes and allied natural products. Prog. Lipid Res..

[14] Jung J.K., Lee S.U., Kozukue N., Levin C.E., Friedman M. (2011). Distribution of phenolic compounds and antioxidative activities in parts of sweet potato (*Ipomoea batata* L.) plants and in home processed roots. J. Food Comp. Anal..

[15] Chien S.C., Young P.H., Hsu Y.J., Chen C.H., Tien Y.J., Shiu S.Y., Li T.H., Yang C.W., Marimuthu P., Tsai L.F.L. (2009). Anti-diabetic properties of three common *Bidens pilosa* variants in Taiwan. Phytochemistry.

[16] Chiang Y.M., Chuang D.Y., Wang S.Y., Kuo Y.H., Tsai P.W., Shyur L.F. (2004). Metabolite profiling and chemopreventive bioactivity of plant extracts from *Bidens pilosa*. J. Ethnopharma..

[17] Tsai L.C., Wang J.C., Hsieh H.M., Liu K.L., Linacre A., Lee J.C. (2008). Bidens identification using the noncoding regions of chloroplast genome and nuclear ribosomal DNA. Forensic. Sci. Int. Genet..

[18] Iqbal S., Younas U., Chan K.W., Zia-Ul-Haq M., Ismail M. (2012). Chemical composition of *Artemisia annua* L. leaves and antioxidant potential of extracts as a function of extraction solvents. Molecules.

[19] Choi Y., Jeong H.S., Lee J. (2007). Antioxidant activity of methanolic extracts from some grains consumed in Korea. Food Chem..

[20] Ghasemzadeh A., Jaafar H.Z.E., Rahmat A. (2011). Effects of solvent type on phenolics and flavonoids content and antioxidant activities in two varieties of young ginger (*Zingiber officinale* Roscoe) extracts. J. Med. Plants Res..

[21] Tsimogiannis D., Samiotaki M., Panayotou G., Oreopoulou V. (2007). Characterization of flavonoid subgroups and hydroxyl substitution by HPLC-MS/MS. Molecules.

[22] Christensen L.P., Kreutzmann S. (2007). Determination of polyacetylenes in carrot roots (*Daucus carota* L.) by high-performance liquid chromatography coupled with diode array detection. J. Sep. Sci..

[23] Simoes-Pires C.A., Queiroz E.F., Henriques A.T., Hostettmann K. (2005). Isolation and on-line identification of antioxidant compounds from three *Baccharis s*pecies by HPLC-UV-MS/MS with post-column derivatisation. Phytochem. Anal..

[24] Shimoi K., Okada H., Furugori M., Goda T., Takase T., Suzuki M., Hara Y., Yamamoto H., Kinae N. (1998). Intestinal absorption of luteolin and luteolin 7-*O*-L-glucoside in rats and humans. FEBS Lett..

[25] Brand-Williams W., Cuvelier M.E., Berset C. (1995). Use of free radical method to evaluate antioxidant activity. LWT-Food Sci. Technol..

[26] Eklund P.C., Langvik O.K., Warna J.P., Salmi T.O., Willfor S.M., Sjoholm R.E. (2005). Chemical studies on antioxidant mechanisms and free radical scavenging properties of lignans. Org. Bimol. Chem..

[27] Chyau C.C., Ko P.T., Mau J.L. (2006). Antioxidant properties of aqueous extracts from *Terminalia catappa* leaves. LWT-Food Sci. Technol..

[28] Rice-Evans C., Miller N.J. (1994). Total antioxidant status in plasma and body fluids. Method. Enzymol..

[29] Chen Y.H., Chang F.R., Lin Y.J., Wang L., Chen J.F., Wu Y.C. (2007). Wu, M.J. Identification of phenolic antioxidants from Sword Brake fern (*Pteris ensiformis* Burm.). Food Chem..

[30] Floegel A., Kim D.O., Chung S.J., Koo S.I., Chun O.K. (2011). Comparison of ABTS/DPPH assays to measure antioxidant capacity in popular antioxidant-rich US foods. J. Food Comps. Anal..

[31] Bhaumik U.K., Kumar A.D., Selvan V.T., Saha P., Gupta M., Mazumder U.K. (2008). Antioxidant and free radical scavenging property of methanol extract of *Blumea lanceolaria* leaf in different *in vitro* models. Pharmacologyonline.

[32] Qiang Z., Ye Z., Hauck C., Murphy P.A., McCoy J.A., Widrlechner M.P., Reddy M.B., Hendrich S. (2011). Permeability of rosmarinic acid in *Prunella vulgaris* and ursolic acid in *Salvia officinalis* extracts across Caco-2 cell monolayers. J. Ethnopharmacol..

[33] Walgren R.A., Walle U.K., Walle T. (1998). Transport of quercetin and its glucosides across human intestinal epithelial Caco-2 cells. Biochem. Pharmacol..

[34] Polli J.W., Wring S.A., Humphreys J.E., Huang L., Morgan J.B., Webster L.O., Serabjit-Singh C.S. (2001). Rational use of *in vitro* P-glycoprotein assays in drug discovery. J. Pharmacol. Exp. Ther..

[35] Vaidyanathan J.B., Walle T. (2001). Transport and metabolism of the tea flavonoid (−)-epicatechin by the human intestinal cell line Caco-2. Pharm. Res..

[36] Silvia Taga M., Miller E.E., Pratt D.E. (1984). Chia seeds as a source of natural lipid antioxidants. J. Am. Oil Chem. Soc..

[37] Zhishen J., Mengcheng T., Jianming W. (1999). Research on antioxidant activity of flavonoids from natural materials. Food Chem..

[38] Shimada K., Fujikawa K., Yahara K., Nakamura T. (1992). Antioxidative properties of xanthone on the auto oxidation of soybean in cylcodextrin emulsion. J. Agric. Food Chem..

[39] Arnao M.B., Cano A., Hernandez-Ruiz J., Garcia-Canovas F., Acosta M. (1996). Inhibition by l-ascorbic acid and other antioxidants of the 2.2’-azino-bis(3-ethylbenzthiazoline-6-sulfonic acid) oxidation catalyzed by peroxidase: A new approach for determining total antioxidant status of foods. Anal. Biochem..

[40] Oyaizu M. (1986). Studies on products of browning reactions: Antioxidant activities of products of browning reaction prepared from glucosamine. Japan J. Nutr..

[41] Søltoft M., Eriksen M.R., Träger A.W.B., Nielsen J., Laursen K.H., Husted S., Halekoh U., Knuthsen P. (2010). Comparison of polyacetylene content in organically and conventionally grown carrots using a fast ultrasonic liquid extraction method. J. Agric. Food Chem..

[42] Zhou S., Feng X., Kestell P., Paxton J.W., Baguley B.C., Chan E. (2005). Transport of the investigational anti-cancer drug 5,6-dimethylxanthenone-4-acetic acid and its acyl glucuronide by human intestinal Caco-2 cells. Eur. J. Pharm. Sci..

[43] Madgula V.L., Avula B., Choi Y.W., Pullela S.V., Khan I.A., Walker L.A., Khan S.I. (2008). Transport of *Schisandra chinensis* extract and its biologically-active compounds across Caco-2 cell monolayers—an *in vitro* model of intestinal transport. J. Pharm. Pharmacol..

